# tiReaction Diffusion Voronoi Diagrams: From Sensors Data to Computing

**DOI:** 10.3390/s150612736

**Published:** 2015-05-29

**Authors:** Alejandro Vázquez-Otero, Jan Faigl, Raquel Dormido, Natividad Duro

**Affiliations:** 1 Department of Computer Sciences and Automatic Control, UNED, C/ Juan del Rosal, 16, Madrid 28040, Spain; E-Mails: raquel@dia.uned.es (R.D.); nduro@dia.uned.es (N.D.); 2 Institute of Physics ASCR, Na Slovance 2, 18221 Prague 8, Czech Republic; 3 Department of Computer Science, Faculty of Electrical Engineering, Czech Technical University in Prague, Technicka 2, 16627 Prague 6, Czech Republic; E-Mail: faiglj@fel.cvut.cz

**Keywords:** reaction diffusion; FitzHugh, Nagumo; path planning; navigation; exploration; Voronoi diagram; laser range sensor; sonar sensor; Turing instability

## Abstract

In this paper, a new method to solve computational problems using reaction diffusion (RD) systems is presented. The novelty relies on the use of a model configuration that tailors its spatiotemporal dynamics to develop Voronoi diagrams (VD) as a part of the system's natural evolution. The proposed framework is deployed in a solution of related robotic problems, where the generalized VD are used to identify topological places in a grid map of the environment that is created from sensor measurements. The ability of the RD-based computation to integrate external information, like a grid map representing the environment in the model computational grid, permits a direct integration of sensor data into the model dynamics. The experimental results indicate that this method exhibits significantly less sensitivity to noisy data than the standard algorithms for determining VD in a grid. In addition, previous drawbacks of the computational algorithms based on RD models, like the generation of volatile solutions by means of excitable waves, are now overcome by final stable states.

## 1. Introduction

Many systems in nature show organization. For example, think about the prenatal development of human embryos or the resource tracking in a population of insects. However, as this organization happens in a spontaneous, way we usually talk about self-organization. General rules under which such structures appear or predictions of their changes are some of the interests of the self-organization field. An approach to model these behaviors relies in the so-called reaction diffusion (RD) processes [1], the Turing instability [2] being a classical method for a stable pattern formation. The study of the complex phenomena modeled by such RD systems has been in the spotlight of the scientific community for a long time, becoming an endless source of attempts for finding out how to take advantage of their rich behavior [3–6]. Computationally, the apparent decision-logic exhibited by the model dynamics can be seen as an abstraction layer for problem solving, allowing one to embed the complexity of a real problem into the model spatiotemporal evolution. This analogy confers the RD model the status of a computational framework in which geometrical operations can be performed. In this paper, we introduce a model setup that exhibits a construction of the Voronoi diagram, considered as a fundamental geometrical structure that can be used to computationally reproduce many natural formations, like soap bubbles [7], bone cells [8] and pattern formation in some mammalian models [9].

Having a set of seeds, the Voronoi diagram can be imagined as partitioning of a plane into a set of cells, where each cell is originated from a particular seed, and the cell represents the region for which the seed of the cell is the closest seed [10]. In other words, the boundary of the regions is formed from points that are equidistant to the two closest seeds. In robotics, the generalized Voronoi graphs (GVG) are used to create a navigational roadmap and a topological representation of the environment [11]. Herein, the equidistant property of the boundary of the Voronoi regions is utilized to find a path that maximizes the clearance from the obstacles. Moreover, the junction places, *i.e.*, locations where the boundary of the cells are connected, represent topologically important places of the environment, like corridor intersections.

The problem being addressed in this paper is to determine the junction places in a grid map of the environment by generation of the Voronoi diagram in the grid map. The proposed RD-based computational model is employed in the generation of such Voronoi diagrams from which the junction places are extracted. We also compare the proposed approach with the state-of-the-art algorithm introduced by the authors of [12]. Based on the presented results, it can be observed that the proposed RD-based computational model is less sensitive to noise than the thinning approach [12]. This new feature also extends the applicability range of our previous works [13–15], where a modelization for tackling robotics problems, like path planning and the exploration task, has been developed.

The paper is organized as follows. The next section provides the theoretical basis of the RD systems and, more particularly, an explanation about how the external (*i.e.*, sensory) information can be introduced into the model dynamics. Section 3 introduces a standard Voronoi algorithm, intended to provide a suitable comparison method for the RD-based approach. Sections 4 and 5 present experimental results and the discussion about the insights found. This paper concludes in Section 7 with a summary of the contributions and conclusions, as well as our future directions.

## 2. Reaction Diffusion Computational Model

### 2.1. The Reaction Diffusion Model of FitzHugh–Nagumo

**Figure 1:**
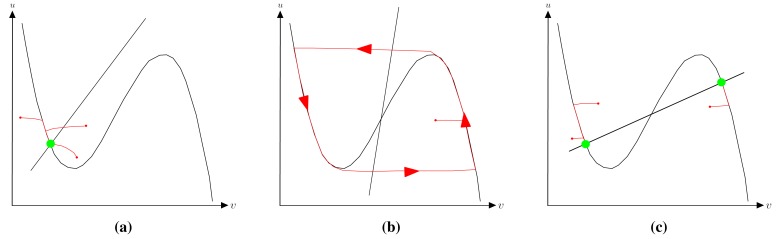
An overview of different nullcline configurations. The green discs represent stable states. Notice that the oscillatory configuration does not have any stable point. The red lines indicate the associated trajectories in the phases space for the particular initial condition. (**a**) Excitable system; (**b**) oscillatory system; (**c**) bistable system.

**Figure 2:**
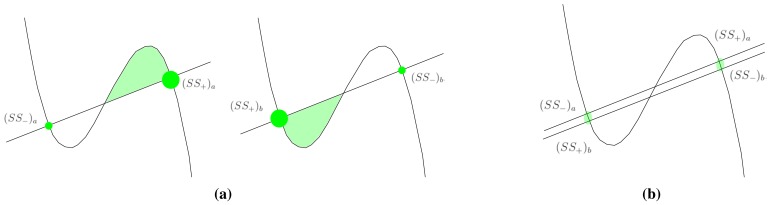
Asymmetric nullcline configurations for a bistable system. The green discs represent stable states. In a spatially extended system, the stable states drive the system to spatially distributed steady states. (**a**) Two possible asymmetric configurations for a bistable system; (**b**) Superposition of both asymmetric configurations.

One of the first reaction diffusion (RD) models was introduced in a seminal work of Hodgkin and Huxley [16] in 1952, where the authors model the electric signaling of firing of individual neurons. Later on, a simplified model was introduced by FitzHugh and Nagumo (FHN) [17,18]. This model adopts the basic form of a coupled system described by:

(1) $$\begin{array}{l} \dot{u}=g\left(u,v\right)+D_{u}\Delta u\\ \dot{v}=f\left(u,v\right)+D_{v}\Delta v \end{array}$$

where *g*(*u, v*) = *ε* (*u* − *u*^3^ − *v* + *φ*) and *f*(*u, v*) = (*u* − *αv* + *β*). Computationally, it describes the evolution of the states variables *u* and *v* for each point over an integration grid, the remaining parameters being constants of the model. The grid points (also called grid cells) are spatially coupled with their neighbors by means of the Laplacian component, which is also responsible for spreading out information along the integration domain. A standard method for analyzing the associated dynamics of Equation (1) involves plotting the nullclines (sometimes called zero-growth isoclines) that describe the dynamics of a zero-dimensional system or a single cell. Thus, for a system characterized by the FHN model, the dynamics of each cell is governed by its associated nullcline configuration, plus the spatial interactions with other cells due to the diffusive coupling. The possible nullcline combination regarding the FHN model is depicted in Figure 1. In particular, Figure 1a corresponds to the aforementioned excitable system in which the nullclines define one single point that is stable. Any initial configuration far from this point will decay to the stable state by means of an excitable wave. The next figure represents an oscillating system that leads to an oscillatory regime. For the purpose of this work, we are interested in the bistable regime depicted in Figure 1c showing a symmetric nullcline configuration. In this configuration, the nullclines define two stable points (drawn as green discs) that are equally stable. Consequently, the concentration levels of the state variables can be found along the integration domain in any of both stable states, where even a heterogeneous combination of both steady states is feasible. Conversely, asymmetric configurations as depicted in Figure 2a lead to one of the stable states possessing a higher stability than the other. In this case, as the natural tendency of the system is evolving towards the concentration level of the most stable state, any initial configuration that is far from this point will inevitably move the whole system to the more stable point. This change is driven by a wavefront, characterized by its strong nonlinear properties. Wavefronts with radically different behaviors can be reproduced in a bistable configuration while maintaining the global behavior of evolving towards the more stable state only by a slight change of the FHN model parameters.

Examples of such a behavior can be found in [13,14], where a non-annihilation behavior for wavefronts was introduced. This property of non-annihilation of the wavefronts allows an implementation of a path planning algorithm that is solely based on the *RD*model to perform the required computations. Later on, a fluid-like behavior was successfully applied in different stages of a robot exploration task [15]. In this paper, we present a behavior of the RD model that provides a computation of Voronoi diagrams.

### 2.2. The Switch-of-Phase Strategy

The nullcline configuration depicted in the left part of Figure 2a characterizes a system with a tendency to evolve towards the stable point (*SS*_+_)*_a_*, while for the right diagram in the same figure, the tendency is towards (*SS*_+_)*_b_*. From a superimposition of the both figures, as is shown in Figure 2b, it is evident that the stable points are relatively close in both nullclines, *i.e.*, (*SS*_−_)*_a_* ≈ (*SS*_+_)*_b_* and (*SS*_+_)*_a_* ≈ (*SS*_−_)*_b_*. The fact that their concentration levels are so close enables a smooth transition from the nullcline configuration depicted in the left part of Figure 2a to the configuration depicted in the right part of the figure, and *vice versa*. Therefore, if the parameters of the model are slightly modified, after a short period of conditioning in which (*SS*_−_)*_a_* becomes (*SS*_+_)*_b_* and (*SS*_+_)*_a_* becomes (*SS*_−_)*_b_*, the model evolves according to the new nullcline configuration. It is because the system dynamics can be tailored to reproduce different behaviors using diverse nullcline configurations that geometric operations like the Voronoi reconstruction can be encapsulated in the model spatiotemporal evolution using this switch-of-phase mechanism. Hence, the problem boils down to finding proper nullcline configurations capable of reproducing the desired behaviors. In the present study, such a behavior is the computation of the Voronoi diagram.

### 2.3. Sensory Information and the External Forcing

It has been shown in the literature that some natural phenomena can be explained in terms of the RD systems, e.g., a pattern formation where self-organization plays a key role. An intriguing characteristic of such natural processes is how their development becomes fairly robust against the unavoidable imbalances that arise in every living system. This behavior is consistently reproduced by the RD dynamics, which exhibits a strong capacity to deal with external perturbations, either overcoming them or adapting its internal behavior to accommodate them. The so-called external forcing has been systematically studied as a mechanism for interacting with RD systems. It comprises both the spatial [19,20] and the spatiotemporal [21] domains, and it can consist of a weak influence that barely affects the dynamics or a strong perturbation that determines its behavior. For perturbing the FHN model as it is defined in Equation (1), the main technique consists of the local modification of the nullcline configuration for a few cells of interest. The effect of this modification on the dynamics is an inhibition of the wavefront propagation at these points; hence, it is equivalent to the introduction of binary information into the system, which can be used to represent obstacles in robot path planning, as was shown in [14]. Besides, the model allows one to consider an additional constraint in the form of an extra (point dependent) term, the final expression to be discretized being:

(2) $$\begin{array}{l} \dot{u}=g\left(u,v\right)+D_{u}\Delta u\\ \dot{v}=f\left(u,v\right)+F+D_{v}\Delta v \end{array}$$

For our interest, the *F* term (*F_ij_* for a cell at the coordinates *ij* in the computational grid) represents the environmental information coming from sensors, and the main difference with regards to the previous method (the local nullcline modification) is that it allows one to introduce gradient-like information. In other words, the FHN dynamics is determined by a combination of the nullcline configuration plus the diffusive term, and such a behavior is somehow resistant to a set of values that can be introduced through *F_ji_* at each cell. For example, it can establish an effective range *F_ji_* ⊂ [0, *max*), which introduces a gradual delay in the wavefront propagation, reaching complete inhibition. An intensive use of this feature is shown in [15], where the RD model is employed for planning a path in the robotic exploration task.

**Figure 3:**
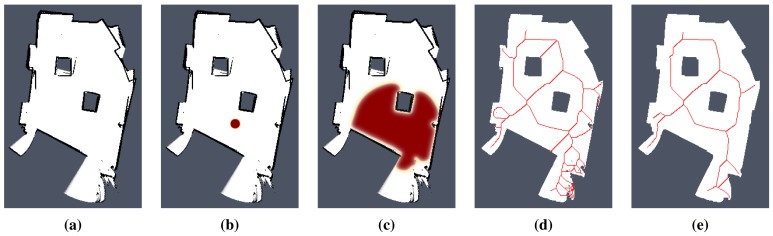
A visualization of sensor measurements transferred to the integration grid of the FHN model for the environment called *cube* and examples of conventional Voronoi diagrams: (**a**) occupancy grid with integrated sensor measurements where detected obstacles are in black, identified freespace is in white, and unknown parts of the environment are in gray; (**b,c**) the occupancy grid that represents the map of the environment is transferred (merged) to the integration grid of the model, where a wavefront is triggered; (**d**) conventional Voronoi diagram in the rough map; (**e**) conventional Voronoi diagram in the map after removing noisy measurements by application of dilation and erosion operations of the mathematical morphology.

An example of an environment representation created from raw sensor measurements is shown in Figure 3a. The map shown is created by integration of the sensors' measurements into an occupancy grid, where each cell denotes a probability that the cell is occupied by an obstacle. New sensor measurements, in this case, a scan from a laser ranger finder, are integrated into the occupancy grid using Bayes' rule [22]. Then, a grid map of the environment is created from the occupancy grid by thresholding the probability value of each cell into one of three states: occupied, free and unknown.

A wavefront evolution in a grid map is shown in Figure 3b,c, where it can be noticed how the wavefront covers the available space during its evolution, while stopping at the edges of the grid map, which represent obstacles. This feature allows one to integrate the external (environmental) information into the RD computational grid in a straightforward way.

## 3. Voronoi Diagram

### 3.1. Conventional Computation of Voronoi Diagrams

**Figure 4:**
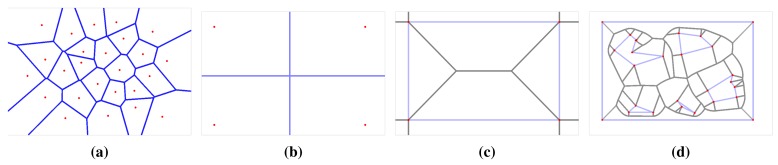
Examples of the ordinary Voronoi diagram for *k* generating points in a plane and generalized Voronoi graph in walled environment: (**a**) random points, *k* = 25; (**b**) four points forming a rectangle, *k* = 4; (**c**) four walls forming a rectangular environment; (**d**) rectangular environment with fives polygonal obstacles with pruned parts of the Voronoi diagram outside the freespace of the polygonal environment.

A computation of a Voronoi diagram (VD) is a basic problem studied in computational geometry. One of the simplest cases of a Voronoi diagram is a division of a plane with *n* points (called generating points) into a set of convex cells, where each cell contains exactly one generating point. For all points inside a cell, the distance to its generating point is smaller than to any other [10]. An example of this ordinary Voronoi diagram is shown in Figure 4a,b.

Many applications of the Voronoi diagram can be found. We are interested in an identification of the topology of the environment to improve the navigation of a mobile robot that is not solely based on metric information, but rather on identification of important places, e.g., crossings and rooms. In our case, the environment is represented by a discrete cell lattice called a grid map. The map can be created from the occupancy grid in which new sensor measurements of the robot's surroundings taken by sonar or laser-based ranger finder are integrated using Bayes' rule [22]. A cell in the occupancy grid has associated probability that the cell is occupied, and thus, a grid map is created from the occupancy grid by thresholding the value of the probability, e.g., all cells with the probability of being occupied higher than 0.5 are considered as obstacles.

Regarding the navigation, a generalized Voronoi graph (GVG) has been introduced in [11] to identify topological places and also to determine paths between the places. A formal definition of the GVG can be found in [23], but basically, the GVG is a one-dimensional set of points that are equidistant to the *n* closest obstacles in *n* dimensions. For a walled environment [12], for example as visualized in Figure 4c,d, the GVG results in a set of points equidistant from two or more obstacles. It is known that Voronoi diagrams are sensitive to noise, which can be seen in Figure 3d,e. That is why Beeson *et al.* introduced an additional pruning technique to remove poor junction places [12]. In this paper, we consider RD-based computation of the Voronoi diagram to determine a skeleton of the grid map that represents the topology of the robot environment. The method is described in the next section.

### 3.2. RD-Based Computation of Voronoi Diagrams

The pioneering work of developing algorithms for computing planar Voronoi diagrams (PVD) using RD models (to the best of our knowledge) deals with the cellular automaton (CA) modelizations of RD systems [24,25] in which some of the well-known RD behaviors were successfully reproduced. In particular, a type of excitable wave that travels and interacts with others, annihilating after a collision, constituted the early pillars for developing RD-based computation. These waves in a cellular automaton lattice spread with a constant velocity, whereby two wavefronts starting from two different sources meet at equidistant points at the same time. The result of the mutual wavefront annihilation at those points is a series of bisectors that separate the wavefront sources. Therefore, it provides a generation of the Voronoi diagram of the sources.

On the experimental side, the so-called RD computers are implemented in a spatially-extended chemical medium, where diffusive or excitation waves propagate and interact. In this way, the results of the computations are represented by spatial distributions of reagent concentrations. Some interesting, yet computationally-limited experimental realizations can be found in [26,27]. These are based on the palladium processor and on a supersaturated solution of sodium acetate, commonly called hot ice. Systematic studies have highlighted the drawbacks of such implementations, like the impossibility of inverting a VD using an RD chemical processor. This characteristic has been shared by all subsequent implementations of experimental RD processors. For instance, VD irreversibility is found in [28].

Although the RD computation is the common substrate for most of these prototypes of processors, they are better contextualized as unconventional computing approaches. These approaches do not only cover RD systems, but they also include a wider scope, like the true slime mold *Physarum polycephalum* [29], a single-celled organism visible to the naked eye that in the plasmodium stage grows up to the square meter scale in size. A comprehensive description of such unconventional methods, including gas discharge, can be found in [30].

### 3.3. FHN-Based Voronoi Diagrams

**Figure 5:**
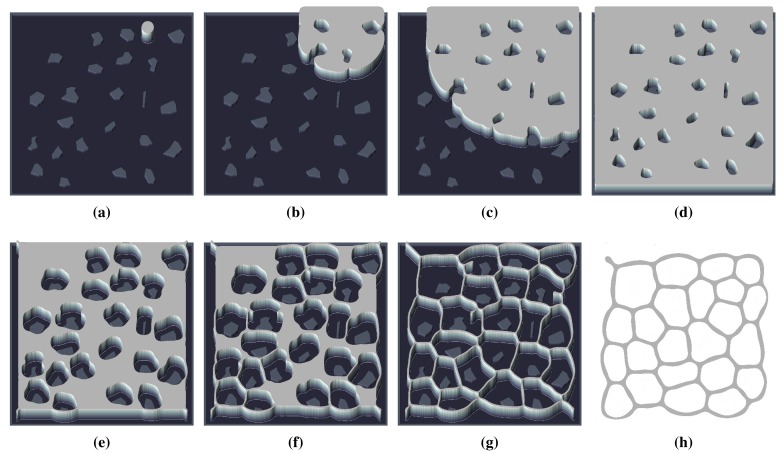
Steps of the RD-based Voronoi algorithm applied to the *potholes* environment: (**a–d**) expansion phase, a wavefront using the *fluid-like* behaviour evolves over the integration domain; (**e–h**) contraction phases, the resulting concentration profile of the previous stage evolves till generating a VD of the environment.

The technique for generating VD by means of the FHN model uses the switch-of-phase mechanism described in Section 2.2, in addition to the way of introducing information in the integration grid as described in Section 2.3. Firstly, an expansion phase that uses the fluid-like behavior (a comprehensive description of this behavior can be found in [15]) triggers a wavefront that evolves till it covers the whole domain. This mechanism is exemplified in the first row of pictures depicted in Figure 5. Then, the second stage based on a contraction phase recovers the wavefront over itself, as is shown in the second row of pictures in Figure 5. The resulting concentration profile coincides with a planar Voronoi diagram. Therefore, all of the logic required for a computation of the diagram is transferred to the spatiotemporal dynamics of the FHN model. The set of required values for the used FHN model are presented in Table 1. Notice that the termination condition for both stages is trivially implemented by measuring the total amount of *u* (or *v*) summing over all grid points. A constant value within two different iterations means the system has reached a static situation; hence, the next phase can be triggered.

## 4. Experimental Results

### 4.1. Comparison Method

**Figure 6:**
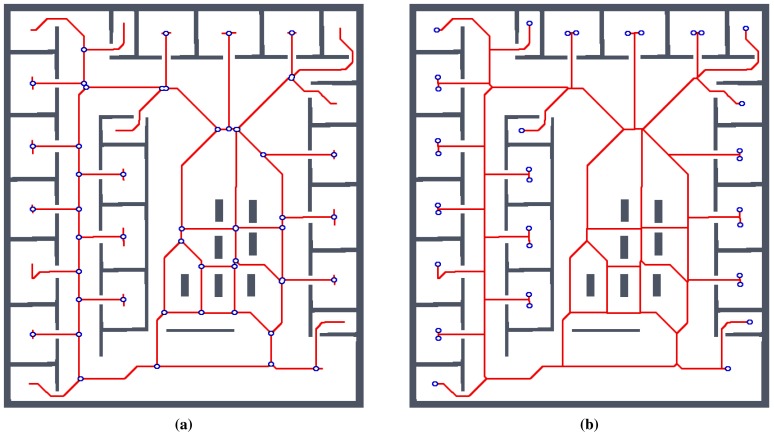
Example of the topology representation of the walled environment *jh* computed as the GVG of the grid map. The computed Voronoi diagram is transformed into a graph, where nodes with the degree (number of incident edges) higher than 2 represent junction places and nodes with the degree 1 are the leaves representing particular rooms. (**a**) junction places; (**b**) leaves.

One of the expected advantages of the proposed RD-based computational model is that it has less sensitivity to spatially noisy data. This is particularly interesting in the application of the Voronoi diagram to compute a skeleton of the grid map of the robot's operational environment that can be used to create a topological representation of the environment. A map of the environment created from the sensor measurements can be noisy, e.g., because of a poor localization, which can lead to a misalignment of the range measurements and a noisy grid map. Regarding the topology, the Voronoi diagram is a graph (skeleton) describing the topology of the environment, where the nodes of the graph represent topologically important places in the environment. The nodes with a degree higher than three are junction places that represent crossings in the environment, while the nodes with a degree of one are the leaves of the skeleton and represent particular areas in the environment, like rooms. A skeleton of a walled environment computed as the Voronoi diagram (by means of the thinning alrotithm [12]) in the grid map of the environment called *jh* is shown in Figure 6, together with the highlighted junction places and leaves that represent rooms.

If we consider a static environment, its topology is fixed, and thus, the number of junction places and leaves should be the same even for an imperfect reconstruction of the map using noisy sensor measurements. Based on this observation, we propose the following methodology to study the sensitivity of the computational model to the noisy sensor data and compare the proposed RD-based Voronoi diagram computation with the thinning algorithm for finding a skeleton of the grid map [12].

**Figure 7:**
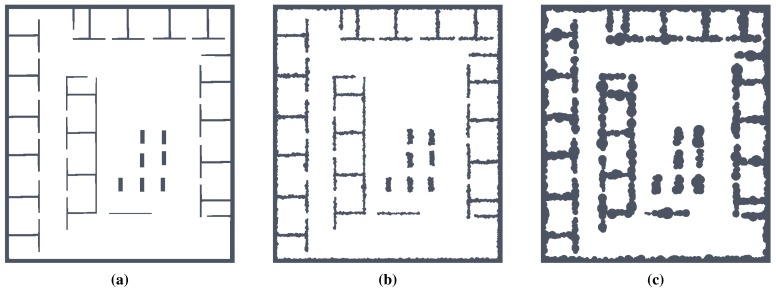
Environment *jh*: (**a**) without added noise; and with added noise for (**b**) *σ_r_* = 2; and (**c**) *σ_r_* = 5.

The evaluation of the sensitivity is based on adding a noise to the map, where the noise is added as small discs with the radius *r* at all boundary cells (*i.e.*, obstacle cells that are incident with at least one free space cell). The radius *r* is drawn from a normal distribution with zero mean and variance *σ_r_* ≤ 10. For each map, we considered 10 values of *σ_r_*, and because the determination of the map skeleton (using the thinning algorithm [12], as well as the proposed RD-based computational model) is deterministic, we create 20 noisy maps with added noise for each original map and a particular value of *σ_r_*. Then, the studied parameters of the skeleton according to the level of noise *σ_r_* are considered as average values and standard deviations computed from 20 trials of each algorithm run on these 20 noisy maps. Having four environments, called *autolab, jh, cube*, and *potholes*, the number of performed trials for two algorithms in this evaluation is 1600. An example of the maps with added noise is shown in Figure 7. Moreover, we also performed additional evaluation for scaled maps for the *jh, cube* and *potholes* environments enlarged by four different scale, which gives 4800 additional trials, which gives 6400 performed trials in total.

The influence of the added noise is studied as the change of the number of detected junction places and the number of leaves according to the reference numbers computed from the noise-less map. The change in the number of junction places is measured as the percentage deviation from the reference value denoted as JPDM. Let *J*_0_ be the number of junction places detected in the noise-less map and *J_σ_* be the number of detected junction places in the map with added noise according to *σ*. Then, the JPDM is computed as:

(3) $$\text{JPDM}=\frac{\bar{J_{\sigma }}-J_{0}}{J_{0}}\cdot 100\%$$

where $\bar{J_{\sigma }}$ is the average number of the detected junction places over all trials for the particular value *σ* and map. The number of leaves is computed as the average number of the detected leaves in the topology graph of the Voronoi diagram that is accompanied by the standard deviation.

A value of the JPDM around zero indicates that the particular method is not sensitive to noise. A negative value indicates that the number of junction places in the noisy map is lower than the reference value *J*_0_. Notice that this does not necessarily mean a worse result. A lower number of junction places is usually related to a lower number of leaves (e.g., detected rooms), and with increasing level of noise, an entrance to a particular room can be more and more narrow. Hence, a robot cannot safely navigate to such a location, and therefore, the room is unreachable; the corresponding leaf is not part of the skeleton, and also, the number of junction places is lower. On the other hand, if the method of the determination of the Voronoi diagram is sensitive to noise, many small branches can be part of the skeleton, and thus, also, the number of junction places is increased.

#### 4.1.1. Determine Topology Graph for the RD-Based Voronoi Diagram

In Figure 5, it can be seen that the skeleton of the map determined by the proposed RD-based computational model is not a set of single pixel width paths, and therefore, there can be various ways to determine the studied indicators.

**Figure 8:**
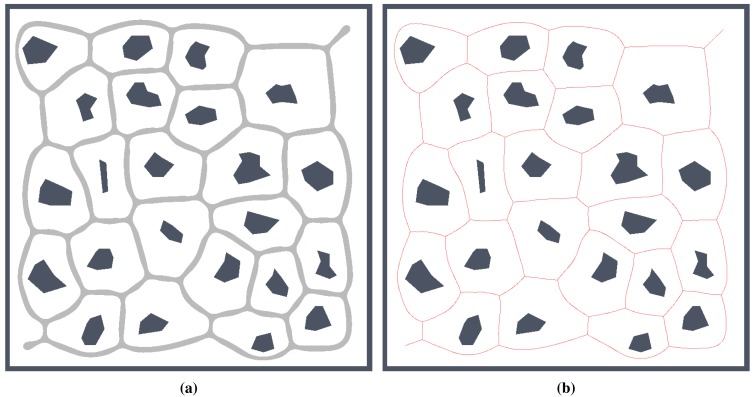
Example of found skeleton (Voronoi diagram) by the RD-based computational model for the pothole environment: (**a**) light gray structure; (**b**) and its one pixel width skeleton shown in red.

**Figure 9:**
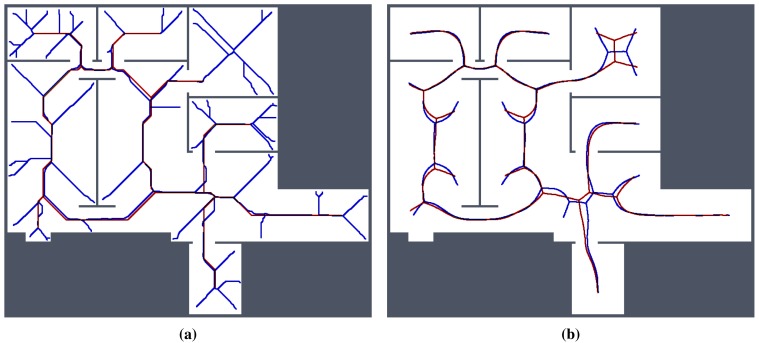

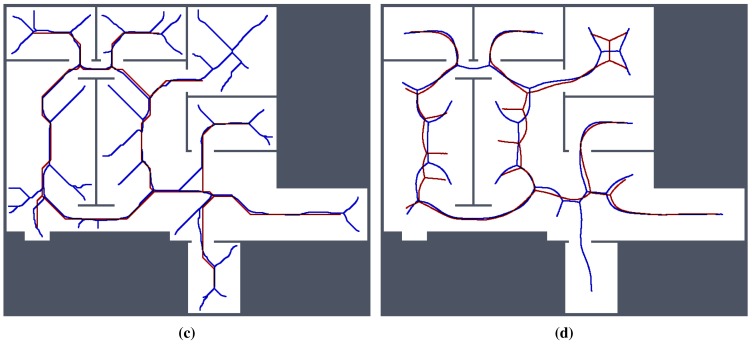
Determined Voronoi diagrams by the thinning and RD-based algorithms in a map of the *autolab* environment for *σ_r_* = 4 and *σ_r_* = 7. The skeleton determined in noise-less map is in red while the skeleton found in the noisy map is in blue. (**a**) Thinning algorithm, *σ_r_* = 4; (**b**) RD-based algorithm, *σ_r_* = 4; (**c**) Thinning algorithm, *σ_r_* = 7; (**d**) RD-based algorithm, *σ_r_* = 7.

For simplicity and also with regard to the used thinning algorithm [12], we considered the RD-based skeleton as a simple polygon (or a set of simple polygons) and determine a single-pixel width skeleton in this polygon using the thinning algorithm. This allows us to consider the identical procedure to compute the indicators for determination of the skeleton based solely on the thinning algorithm and on the RD-based computational model. Such a skeleton of the determined RD-based Voronoi diagram is shown in Figure 8. An example of superimposed Voronoi diagrams determined in the map with and without the added noise in the environment *autolab* is shown in Figure 9.

It can be noticed that the thinning algorithm prunes branches of the skeleton that would go to the corners of the particular rooms. On the other hand, this is not exactly the case of the RD-based computation of the skeleton, which splits the skeleton according to the space around the propagated wavefront. Therefore, instead of direct comparison of the RD-based skeleton with the thinning algorithm accompanied by advanced pruning of branches, we study how the determined skeletons differ with increasing *σ_r_* for each particular method, and thus, we study the algorithms' sensitivity to the level of noise.

#### 4.1.2. Results

**Figure 10:**
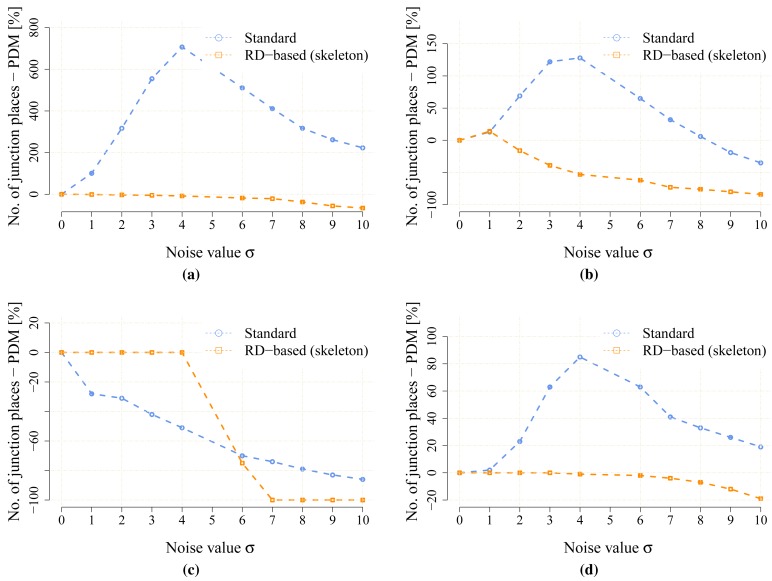
The noise sensitivity indicator JPDM Equation (3) in the noisy maps created for the particular value of the noise level *σ*. (**a**) environment *autolab*; (**b**) environment *jh*; (**c**) environment *cube*; (**d**) environment *potholes*.

**Figure 11:**
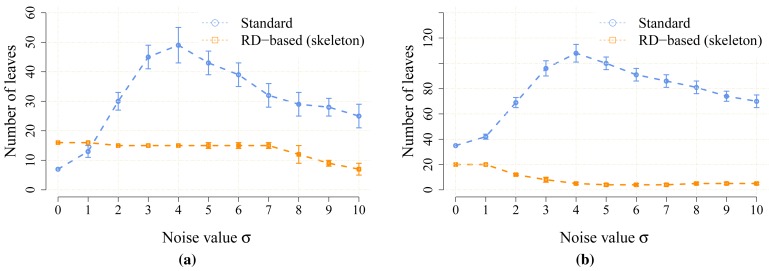

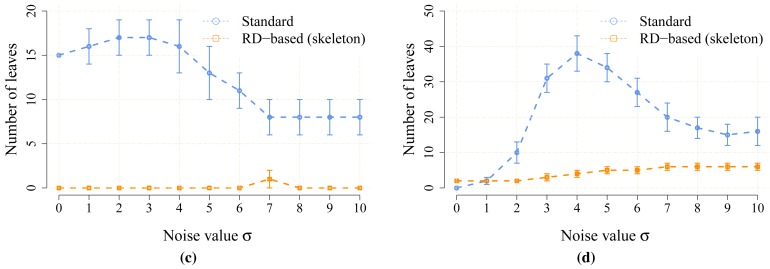
Average numbers of the leaves detected in the noisy maps created for the particular value of the noise level *σ*. (**a**) environment *autolab*; (**b**) environment *jh*; (**c**) environment *cube*; (**d**) environment *potholes*.

**Figure 12:**
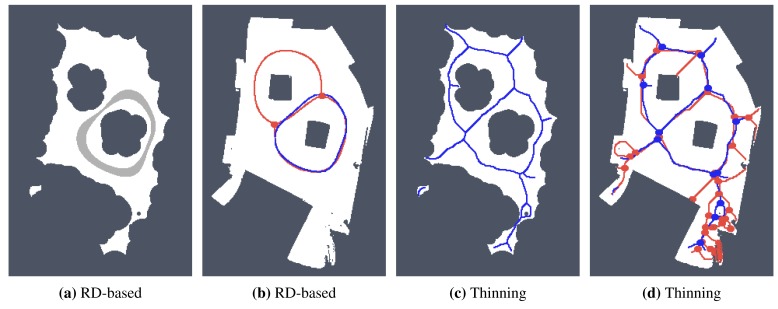
Skeletons and the determined topological maps with highlighted junction places and leaves in the map *cube* with the noise level *σ* = 7: (**a**) RD-based Voronoi diagram; (**b**) and its corresponding skeleton (shown in green) that is superimposed on the skeleton determined in the noise-less map (in red); (**c**) Pruned GVG representing skeleton of the map determined by the thinning algorithm [12]; (**d**) and the corresponding skeleton superimposed on the skeleton determined in the noise-less map.

**Figure 13:**
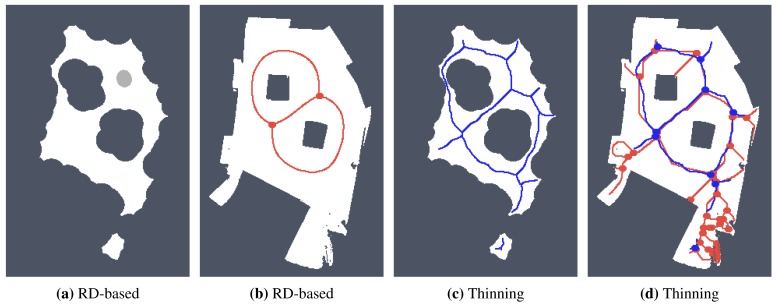
Determined skeletons and topological maps with highlighted junction places and leaves in the map *cube* with the noise level *σ* = 8: (**a**) RD-based Voronoi diagram; (**b**) and its corresponding skeleton (shown in green) that is superimposed on the skeleton determined in the noise-less map (in red); (**c**) Pruned GVG representing skeleton of the map determined by the thinning algorithm [12]; (**d**) and the corresponding skeleton superimposed on the skeleton determined in the noise-less map.

**Figure 14:**
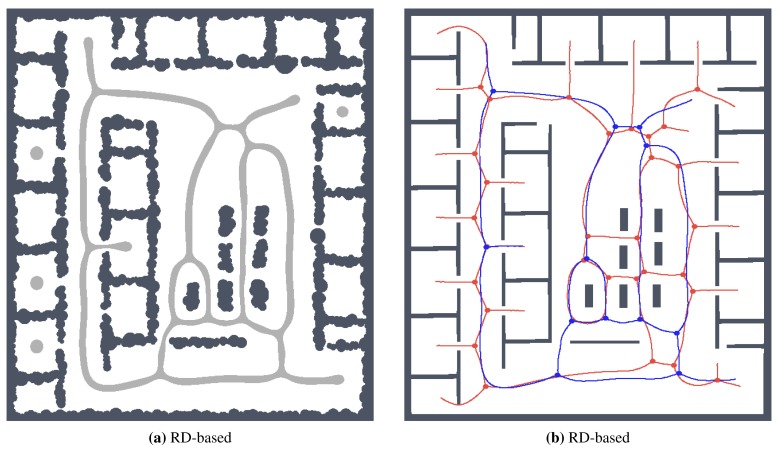

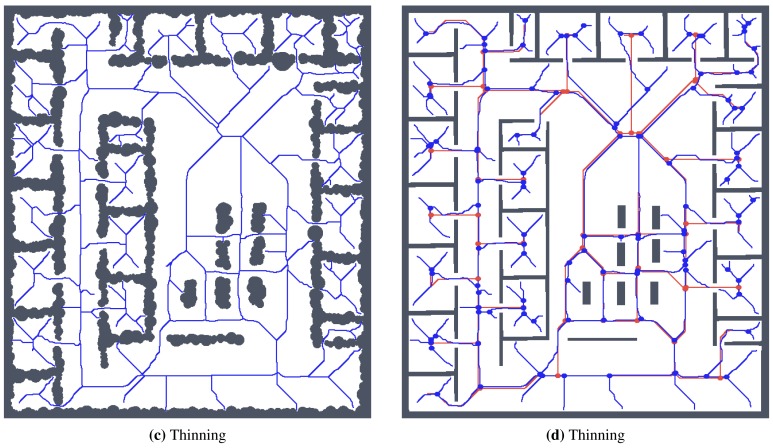
Determined skeletons and topological maps with highlighted junction places and leaves in the map *jh* with the noise level *σ* = 4: (**a**) RD-based Voronoi diagram; (**b**) and its corresponding skeleton superimposed on the skeleton determined in the noise-less map (in red); (**c**) Pruned GVG representing skeleton of the map determined by the thinning algorithm [12]; (**d**) and the corresponding skeleton superimposed on the skeleton determined in the noise-less map.

The proposed statistical indicators of the noise sensitivity, *i.e.*, JPDM Equation (3), and the average number of the detected leaves are depicted in Figures 10 and 11, respectively. The results show that the proposed RD-based computation of the Voronoi diagram is less sensitive to the added noise, as the number of junction places is usually lower than the number of junction places for the map without the noise. The lowest value of the JPDM is –100% for the *cube* environment, where none of the junction places have been detected in the environment, see Figures 12 and 13. On the other hand, the thinning algorithm is a more sensitive to the noise as the number junction places is quickly increasing, and for the *autolab, jh* and *potholes* environments, the maximal number of the junction places is for the noise level *σ* = 4; see the example in Figure 14.

**Figure 15:**
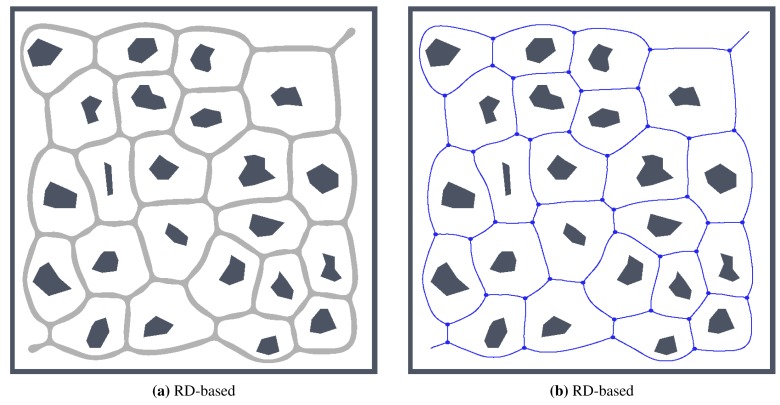

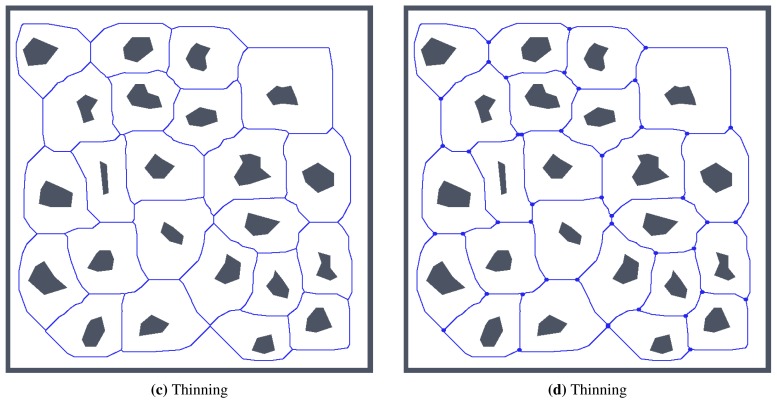
Determined skeletons and topological maps with highlighted junction places and leaves in the map *potholes* with the noise level *σ* = 0: (**a**) RD-based Voronoi diagram; (**b**) and its corresponding skeleton superimposed on the skeleton determined in the noise-less map (in red); (**c**) Pruned GVG representing skeleton of the map determined by the thinning algorithm [12]; (**d**) and the corresponding skeleton superimposed on the skeleton determined in the noise-less map.

**Figure 16:**
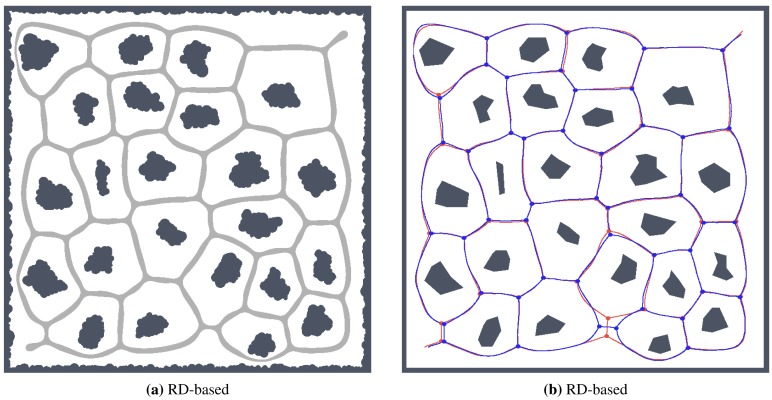

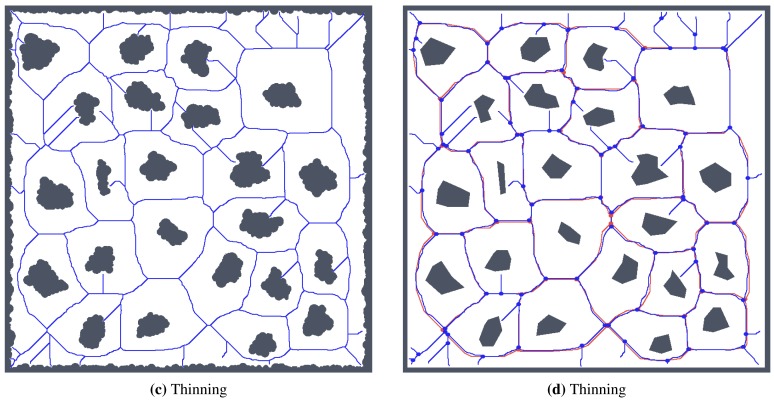
Determined skeletons and topological maps with highlighted junction places and leaves in the map *potholes* with the noise level *σ* = 4: (**a**) RD-based Voronoi diagram; (**b**) and its corresponding skeleton superimposed on the skeleton determined in the noise-less map (in red); (**c**) Pruned GVG representing skeleton of the map determined by the thinning algorithm [12]; (**d**) and the corresponding skeleton superimposed on the skeleton determined in the noise-less map.

The number of leaves significantly changes with the noise level for the thinning algorithm, while for the proposed RD-based computation of the skeleton, it changes only slightly and typically decreases with a higher level of the noise *σ*, as can be seen in Figure 11. The only exception is the environment *potholes*, where the initial skeleton for *σ* = 0 does not contain any leaves; see Figure 15, while with increasing *σ*, some parts become unreachable for the robot (wavefront propagation), which is visualized in Figure 16.

#### 4.1.3. Influence of the Map Resolution

**Figure 17:**
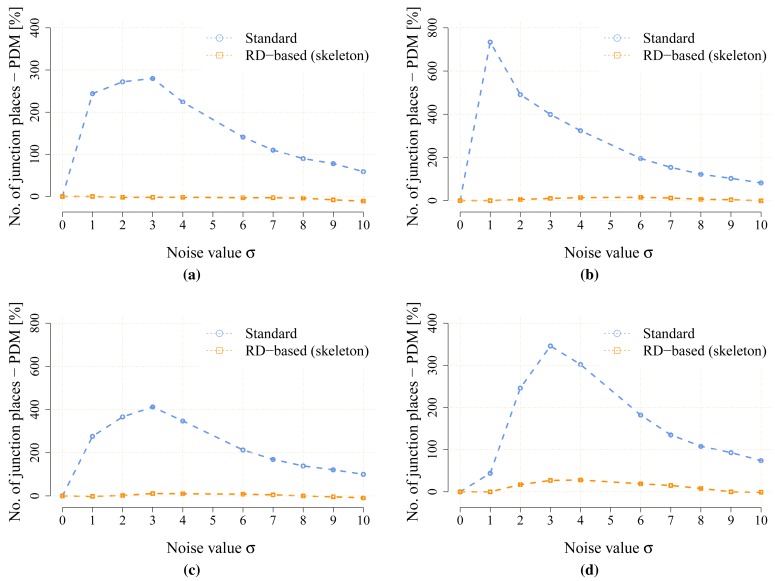
The noise sensitivity indicator JPDM Equation (3) in the scaled noisy maps of the *potholes* environment. (**a**) *potholes*, 960 × 960; (**b**) *potholes*, 1200 × 1200; (**c**) *potholes*, 1360 × 1360; (**d**) *potholes*, 1600 × 1600.

**Figure 18:**
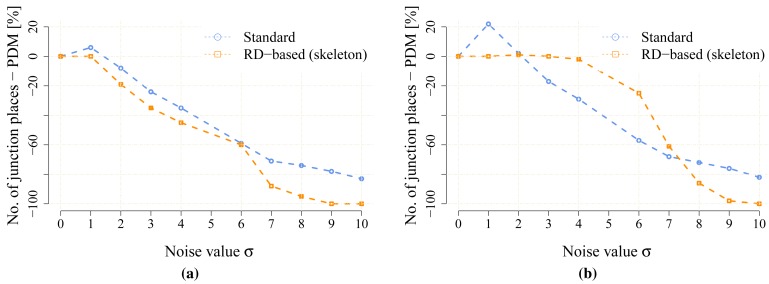

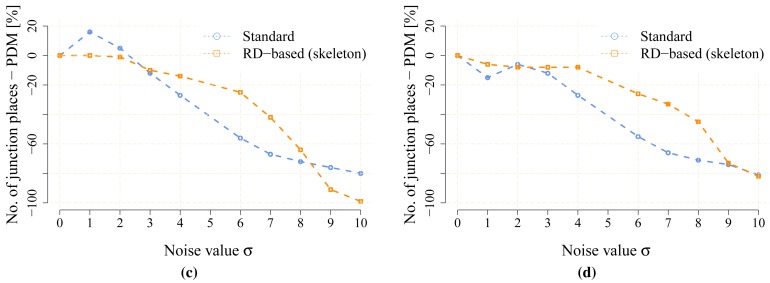
The noise sensitivity indicator JPDM Equation (3) in the scaled noisy maps of the *cube* environment. (**a**) *cube* 283 × 419; (**b**) *cube*, 354 × 524; (**c**) *cube*, 401 × 593; (**d**) *cube*, 472 × 698.

**Figure 19:**
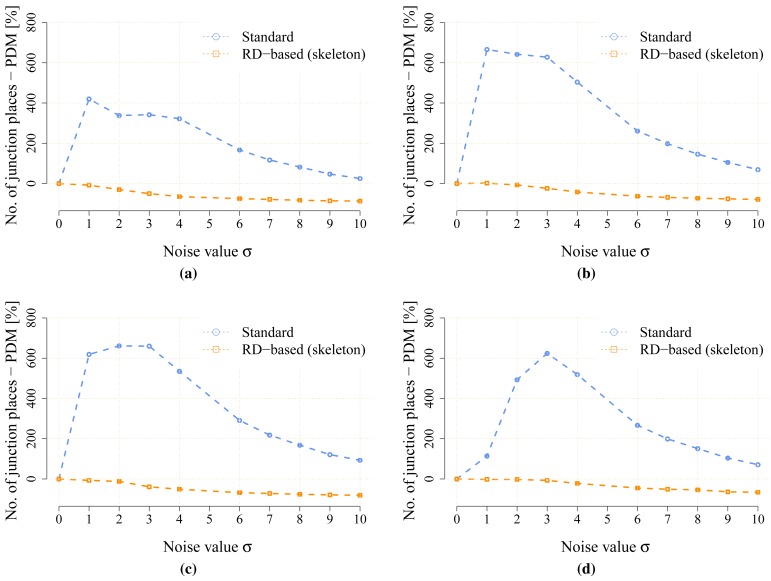
The noise sensitivity indicator JPDM Equation (3) in the scaled noisy maps of the *jh* environment. (**a**) *jh*, 768 × 863; (b) *jh*, 960 × 1079; (**c**) *jh*, 1088 × 1222; (**d**) *jh*, 1280 × 1438.

**Figure 20:**
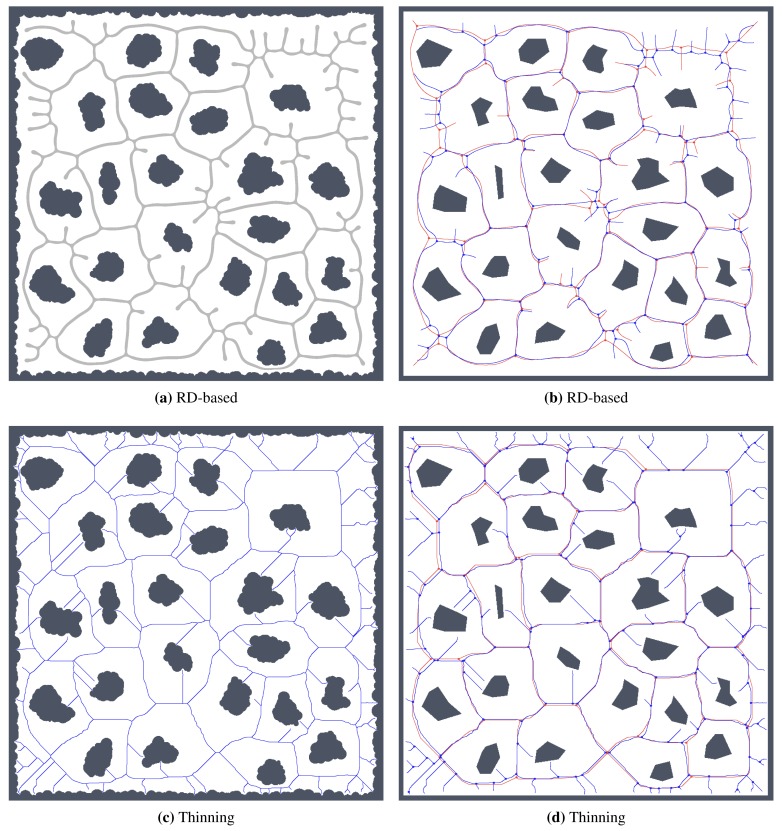
Determined skeletons and topological maps with highlighted junction places and leaves in the map *potholes* with the noise level *σ* = 5 for the scaled map with 1600 × 1600 grid points: (**a**) RD-based Voronoi diagram; (**b**) and its corresponding skeleton superimposed on the skeleton determined in the noise-less map (in red); (**c**) Pruned GVG representing skeleton of the map determined by the thinning algorithm [12]; (**d**) and the corresponding skeleton superimposed on the skeleton determined in the noise-less map.

**Figure 21:**
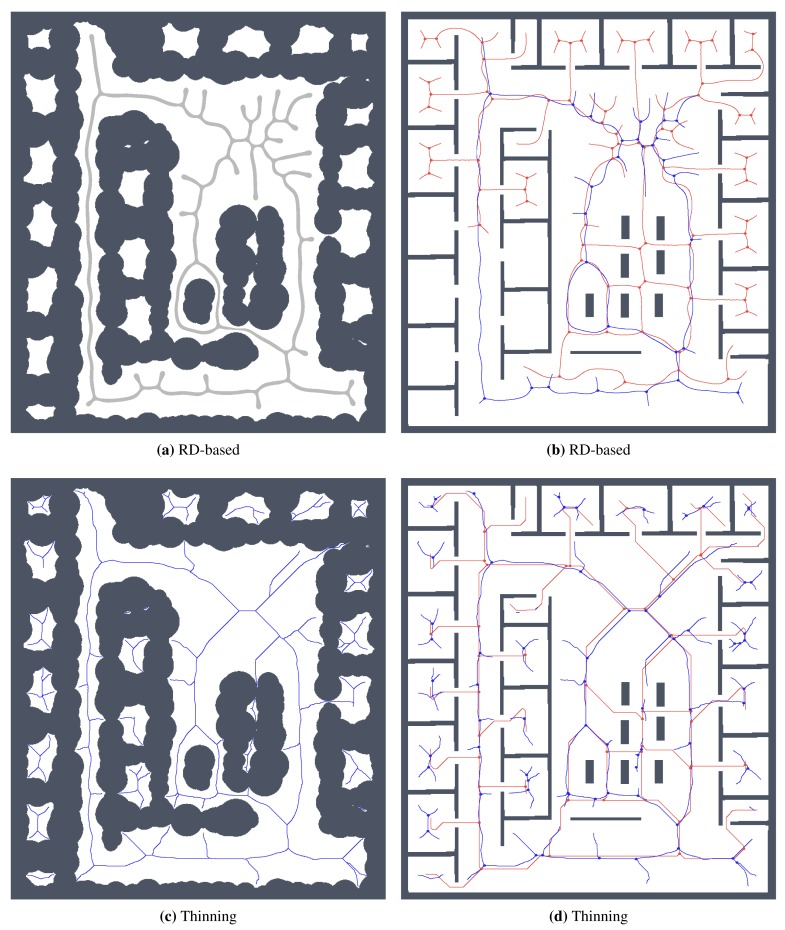
Determined skeletons and topological maps with highlighted junction places and leaves in the map *jh* with the noise level *σ* = 10 for the scaled map with 1280 × 1438 grid points: (**a**) RD-based Voronoi diagram; (**b**) and its corresponding skeleton superimposed on the skeleton determined in the noise-less map (in red); (**c**) Pruned GVG representing skeleton of the map determined by the thinning algorithm [12]; (**d**) and the corresponding skeleton superimposed on the skeleton determined in the noise-less map.

**Figure 22:**
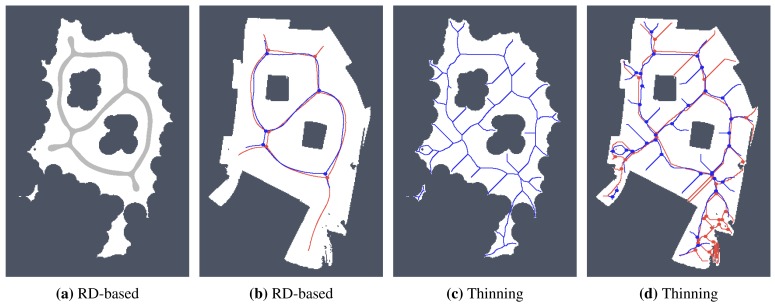
Determined skeletons and topological maps with highlighted junction places and leaves in the map *cube* with the noise level *σ* = 5 for the scaled map with 478 × 698 grid points: (**a**) RD-based Voronoi diagram; (**b**) and its corresponding skeleton superimposed on the skeleton determined in the noise-less map (in red); (**c**) Pruned GVG representing skeleton of the map determined by the thinning algorithm [12]; (**d**) and the corresponding skeleton superimposed on the skeleton determined in the noise-less map.

The resolution of the grid map may influence both computational techniques to determine the Voronoi diagram, and therefore, its influence has been studied for the *potholes, cube* and *jh* environments for which all the maps have been scaled 1.2, 1.5, 1.7 and 2 times. Particular results of the JPDM indicator are shown in Figures 17, 18–19. Examples of the determined skeletons for both algorithms using different map resolutions are shown in Figures 20, 21–22. Although a higher resolution increases the computational burden of both techniques, it can be noticed that the proposed RD-based computation is significantly less sensitive to the change of the resolution, and the number of junction places and leaves remains almost the same according to the change in the thinning algorithm.

## 5. Discussion

The presented evaluation of the Voronoi diagram generation for the maps with added noise demonstrates that one of the main advantages of the RD-based computation is a lower sensitivity to the noise level in comparison to the thinning algorithm. This advantage comes from the stability, in the Lyapunov sense, of the FHN system. The dynamics of the system drive it to the stable state, so that the spatiotemporal evolution is resistant to the inclusion of external information, which in the present case is interpreted as noise. This is mainly visible in the skeleton of the environment, where the RD-based skeleton does not include new branches for small “pockets” created as a result of the added noise. As a consequence, the number of junction places slowly decreases with the increasing noise level, which means that some parts of the environment become unreachable for the robot. On the other hand, the thinning algorithm is sensitive to the added noise, and additional pruning techniques are necessary.

Regarding these observations, the proposed RD-based computational model seems to be a suitable technique to process real sensor data and provides more robust solutions than purely geometrical-based approaches. The RD-based computational model works on a computational grid, where the underlying non-linear dynamics model exhibits its behavior based on the particular system properties. One of the important properties is the resolution of the computational grid itself, which is related to the ability of the wavefront propagation into a narrow passage in the environment. Thus, it is related to the dimension of the robot, and it guarantees finding a safe path. Although an additional pruning of the determined skeleton can be used to guarantee the required clearance of the topological roadmap (*i.e.*, generated skeleton of the environment), the relation is an indivisible part of the RD-based model.

It is noteworthy that this approach takes advantage of the common and well-known properties inherent to RD systems, like the natural parallelism of the computation and fault tolerance to damaged cells, interpreted as noise resistance. Besides, it is possible to physically implement this model by means of already existing technologies, such as CNN [31] via a VLSIchip, a feature that enables real-time computations.

## 6. Comments on the Stability of the RD System and Reduction of Computational Requirements

A computation of the RD-based model evolution can be significantly sped up based on the observation of the system stability in the sense of Lyapunov. The wavefronts whose propagation leads to the shift from one stable state to the other define the only regions in the environment that are actually evolving. In the computational grid, this means that as the evolution of the grid cells reaches the new stable state, these nodes remain in this state unchanged. Hence, this observation can be utilized to decrease the computational burden of the spatial integration of the model by performing computations only for these grid cells. Notice that although this approach can restrict the propagation of the wavefronts in the boundaries of evolving areas, the values of *u* and *v* tend to a stable level, and therefore, the restrictions only limit the velocity of the propagation. Moreover, the velocity of the underlying physical model is also limited and constant, and therefore, using the knowledge of the propagation behavior, one can set suitable parameters without affecting the propagation. These parameters have been experimentally set for the particular developed approach.

The proposed routine can be exemplified as follows: after several computational steps, cells are examined to see if their value of the concentration level *u* has changed after the selected period *T_active_, i.e.*, testing the value *u_activation_* = |*u*(*k*) − *u*(*k*+*T_active_*)| of the computational steps *k* and *k*+*T_active_*. Then, the cells for which the difference *u_activation_* is above a certain limit (e.g., > 10^−3^) are denoted as active for the next batch of the computational steps. Besides, a small neighborhood of each such active cell is also marked as active to further support a propagation of the wavefronts. The herein used discretization of the FHN model is a simple finite difference method on a Cartesian grid: a simple forward in time centered in space (FTCS) scheme that facilitates the manipulation of the integration loop for including the described optimization.

Although a detailed comparison is out of the scope of the present work, a basic comparison for the two environments *potholes* and *jh* is shown in Table 2. Using a grid resolution of 800 × 800 points as a baseline for the *potholes* environment and 840 × 960 for the *jh* environment, four additional resolutions have been computed for the propagation phase of the Voronoi problem. The results show that the proposed optimization technique scales well with the increasing grid resolution. Moreover, since this optimization is hardware-agnostic, it can be ported to specialized platforms.

## 7. Conclusions and Future Work

Computational geometry is concerned with the study of algorithms for solving geometrical problems, and the Voronoi diagram arises as a data structure that underlies the geometry of seemingly different ones. An example of this, in a discrete cell lattice, is the generalized Voronoi graph, which identifies topological places and also determines paths between the places. In that context, the Voronoi diagram can be used for addressing the robot navigation problem.

In this paper, we consider RD-based computation of the Voronoi diagram to determine a skeleton of the grid map that represents a topology of the robot's operational environment. The herein presented approach binds robotics and the Voronoi diagram under a computational umbrella of RD systems, which allows a direct integration of raw sensor data into the computational grid.

The discovered findings are clearly split into two distinctive features: first is the exploitation of the newly-found behavior that leads to Voronoi-like concentration patterns as the final stable states of the model's spatiotemporal evolution, also allowing one to recover the system to its original state by means of the Voronoi diagram inversion; the second feature is the lack of sensitivity to the added noise that makes any pruning technique unnecessary for removing poor junction places, as is required in the case of the considered thinning algorithm.

Furthermore, the presented results are an extension of our previous studies, where different behaviors were successively included in the RD-based computational core and successfully applied to decision-planning and motion-planning in robotics. Therefore, the addition of the Voronoi diagram pattern sets the foundation of a reaction diffusion-based computational framework suitable for addressing geometrical problems, up to now particularly tailored to face the challenges associated with navigational problems in robotics.

## Figures and Tables

**Table 1:** Set of values for the FHN model to reproduce the desired behavior in both phases: expansion and contraction.

		**FHN Parameters**				

		*α*	*β*	*ϵ*	*D_u_*	*D_v_*
**Phase**	*expansion*	4	−0.5	40	0.45	2
	*contraction*	3.8	0.1	10	0.1	1.5

**Table 2:** Computational time (in minutes) spent in the expansion phase of the Voronoi computation for the environments *potholes* and *jh*. For both variants of the RD-based algorithm: the original, named naive, and the optimized version according to Section 6, named optimized. The size of the baseline is 800 × 800 grid points for the *potholes* and 840 × 960 grid points for the *jh*. The computational times have been collected using a workstation with an Intel iCore7 3770 processor, Debian jessie (64-bit), 16 GB RAM and C++ implementation compiled by the GCC 4.9.2 with the O3 enabled.

		**Resolution Factor**				

		***1***	***1.2***	***1.5***	***1.7***	***2***
*potholes*	*naive*	8.49	15.3	27.4	44.4	69.3
	*optimized*	1.3	2.1	2.5	3.3	5.2

*jh*	*naive*	12.5	20.7	44.5	65.8	103.4
	*optimized*	1.4	2.2	4.1	5.2	7.2
